# Prognostic significance of the triglyceride-glucose index for patients with ischemic heart failure after percutaneous coronary intervention

**DOI:** 10.3389/fendo.2023.1100399

**Published:** 2023-02-06

**Authors:** Tienan Sun, Xin Huang, Biyang Zhang, Meishi Ma, Zheng Chen, Zehao Zhao, Yujie Zhou

**Affiliations:** Department of Cardiology, Capital Medical University Affiliated Anzhen Hospital, Beijing, China

**Keywords:** ischemic heart failure, percutaneous coronary intervention, insulin resistance, triglycerideglucose index, adverse cardiovascular events

## Abstract

**Background:**

In previous studies, the TyG index (triglyceride-glucose index) has been proven to be closely associated with the prognosis of cardiovascular disease. However, the impact of TyG index on the prognosis of patients with ischemic HF (heart failure) undergoing PCI (percutaneous coronary intervention) is still unclear.

**Method:**

In this study, 2055 patients with ischemic HF were retrospectively enrolled and classified into four groups based on quartiles of the TyG index. The primary endpoint was MACE (major adverse cardiovascular events) consisting of all-cause mortality, non-fatal MI (myocardial infarction), and any revascularization. The incidence of the endpoints among the four groups was assessed through Kaplan-Meier survival analysis. The independent correlation between TyG index and endpoints was analyzed with multivariate Cox regression models. Besides, the RCS (restricted cubic spline) analysis was performed to examine the nonlinear relationship between TyG index and MACE.

**Result:**

The incidence of MACE was significantly higher in participants with a higher TyG index. The positive association between the TyG index and MACE was also confirmed in the Kaplan–Meier survival analyses. Multivariate cox proportional hazards analysis indicated that the TyG index was independently associated with the increased risk of MACE, regardless of whether TyG was a continuous [TyG, per 1−unit increase, HR (hazard ratio) 1.41, 95% CI (confidence interval) 1.22-1.62, P < 0.001] or categorical variable [quartile of TyG, the HR (95% CI) values for quartile 4 was 1.92 (1.48-2.49), with quartile 1 as a reference]. In addition, the nonlinear association of TyG index with MACE was shown through RCS model and the risk of MACE increased as the TyG index increased in general (Nonlinear p=0.0215). Besides, no obvious interaction was found in the association of TyG with MACE between the DM (diabetes mellitus) group and the no-DM group.

**Conclusion:**

Among patients with ischemic HF undergoing PCI, the TyG index was correlated with MACE independently and positively.

## 1 Introduction

Heart failure is a rapid-growing public health problem estimated to affect more than 37.7 million people worldwide (1). In the coming years, the burden of HF worldwide will increase significantly as the global population ages (2). Ischemic heart disease is the most common cause of HF due to left ventricular dysfunction arising from myocardial ischemia or infarction (3, 4).

As a hallmark of metabolic disorders and systemic inflammation (5), IR (insulin resistance) has been proven to be significantly associated with ASCVD (atherosclerotic cardiovascular disease) and contributes to an adverse prognosis (6–8). Meanwhile, there is also evidence that IR may play an important role in the development and progression of HF. Higher IR levels were associated with a higher risk of developing HF, which has been suggested in studies comprising individuals with or without T2DM (type2 diabetes mellitus) (9).

Hyperinsulinaemic-euglycaemic clamp technique, the gold standard to assess IR, is impractical for large-scale epidemiological and clinical studies due to the complex, expensive, and time-consuming procedure (10, 11). Previous studies have shown that TyG is highly correlated with IR measured by hyperinsulinaemic-euglycaemic clamp, and even performs better than HOMA-IR (homeostasis model assessment of IR), either in individuals with or without T2DM (type2 diabetes mellitus) (10, 12, 13). Subsequently, the relationship between TyG index and many cardiovascular diseases has been demonstrated in many studies (14–17).

However, no research was conducted to investigate the impact of TyG index on the prognosis of patients with ischemic HF undergoing PCI. This study aimed to identify the potential correlations between IR assessed by the TyG index and clinical prognosis in ischemic HF patients undergoing PCI.

## 2 Method

### 2.1 Study population

**Figure 1:**
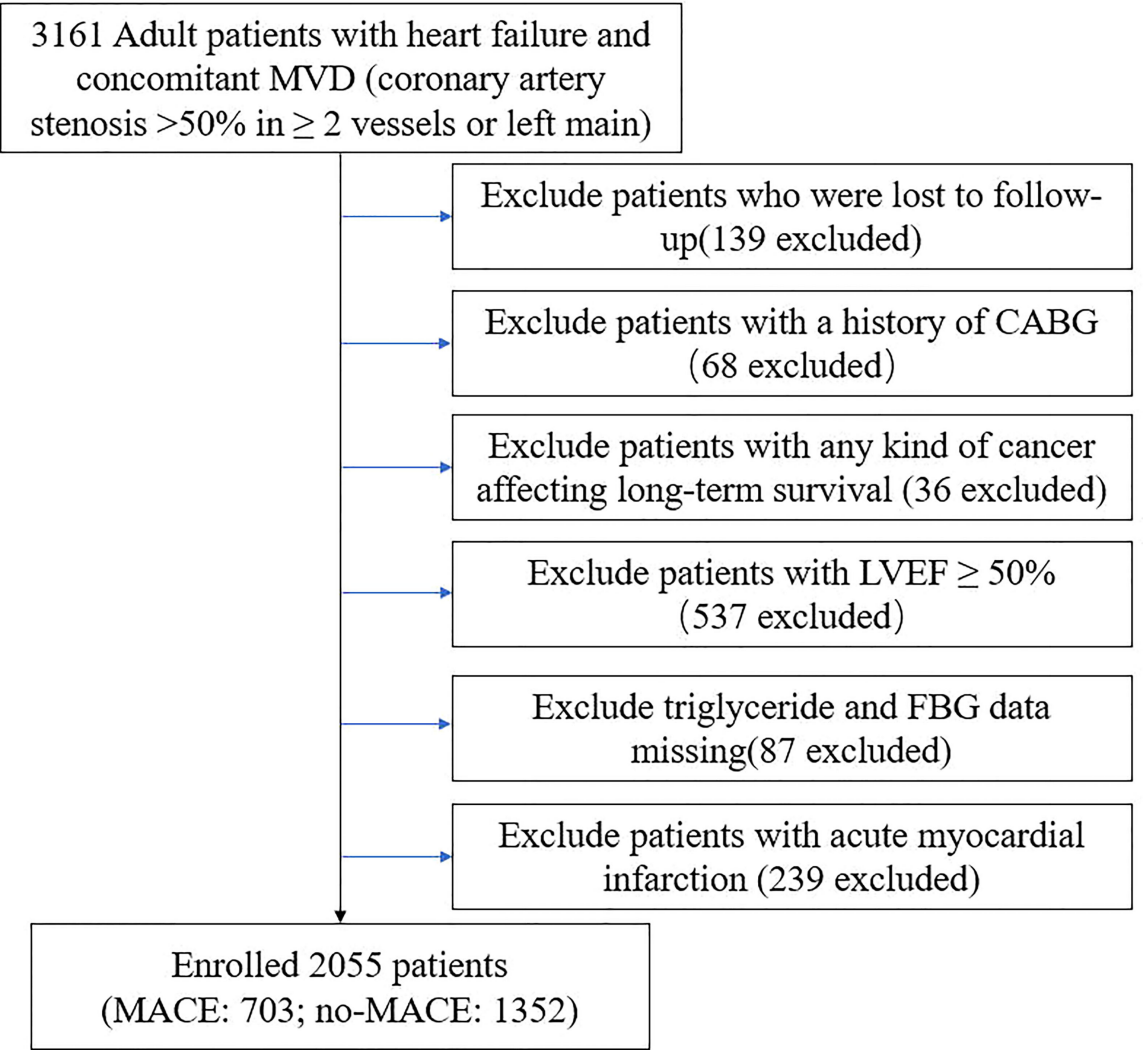
Flow chart of study population.

This study was a single-center, observational, retrospective cohort study, involving ischemic HF patients undergoing elective PCI (June 2017 - June 2019) at Beijing Anzhen Hospital. Patients with ischemic HF were selected according to the following criteria (3): (1) HF diagnosis according to ICD (International Classification of Diseases) 10th revision (Details can be found in **Supplementary**) (2) MVD (concomitant multivessel disease: coronary artery stenosis >50% in >_2 vessels or left main artery disease). A total of 3161 adult patients with ischemic HF undergoing elective PCI in our cardiovascular center were enrolled in this cohort. The exclusion criteria of this study included: (1) patients lost to follow-up (2) history of CABG (coronary artery bypass grafting) (3) any kind of cancer affecting long-term survival (4) LVEF (left ventricular injection fraction) ≥50% (5) TGs and FBG data missing (6) acute MI (7) patients refusing to sign informed consent. Ultimately, 2055 patients were included in the final analysis (**Figure 1**).

### 2.2 Data collection and definitions

Demographics, vital signs, NYHA (New York Heart Association) class, comorbidities, medical history, laboratory parameters, echocardiography, medication, angiographic data, and procedural results were derived from Beijing Anzhen Hospital’s electronic medical record system (Details can be found in **Supplementary**). The coronary artery lesion characteristics were determined by at least two experienced cardiologists after analyzing the angiographic data. PCI was conducted based on the latest practice guidelines in China (18), and precise strategies during PCI were devised by skilled interventional cardiologists. The details of lesion characteristics (19) can be found in the **Supplementary**. SYNTAX score was calculated according to the SYNTAX score algorithm (www.syntaxscore.com) (20).

### 2.3 Follow−up

Participants were routinely followed up by trained professionals at 3, 6, 9, 12, 24, and 36 months after baseline PCI. MACE information was collected *via* telephone questionnaires and outpatient office visits from the patients or their families. Whenever necessary, corresponding medical records were checked to confirm the information.

### 2.4 Grouping and endpoints

The TyG index was calculated using the formula: Ln (fasting TG [mg/dL] × FBG [mg/dL]/2). All patients were stratified into four groups according to TyG quartiles.

The primary endpoint was MACE, which consisted of all-cause mortality, non-fatal MI, and any revascularization. The definition of MI was determined by the fourth universal definition of MI (21). Any revascularization was defined as coronary revascularization due to any reason. And the secondary endpoints were the components of MACE.

Among patients who experienced multiple adverse endpoints over the course of follow-up, the most severe adverse endpoint was selected for analysis (all-cause mortality > non-fatal MI > any revascularization). Only the first occurrence was analyzed when a single event occurred more than once. In the present study, the follow-up lasted until June 2022.

### 2.5 Statistical analysis

Normally distributed variables were presented as mean ± standard deviation (SD) and compared between the groups by means of ANOVA test. Categorical variables were presented as number (percentage) and the Chi-squared test was conducted to compare differences among groups.

An analysis of Cox proportional hazards regression was conducted to estimate the HR and the 95% CI for the primary and secondary observational endpoints. Variables added to the multiple regression were identified through univariate analysis (P< 0.05). The first quartile group of TyG served as the reference group. No variables were adjusted in model 1. In model 2, age and sex were incorporated into the adjustment. In model 3, sex, heart rate, body mass index, NYHA class, prior PCI, albumin, TC(total cholesterol), LDL-C(low-density lipoprotein cholesterol), HDL-C(high-density lipoprotein cholesterol), potassium, uric acid, LVEF, ARB(angiotensin receptor blocker), thiazide diuretics, spironolactone, tolvaptan, sacubitril/valsartan, diffuse lesion, SYNTAX score, LM (left main artery) disease, in-stent restenosis, chronic total occlusion target vessel (LM) were incorporated into the model. In order to assess the incidence rate of adverse events among groups stratified by TyG quartiles, Kaplan–Meier survival analyses were performed and the log-rank test was conducted to determine discrepancies among four quartiles. Subgroup analysis was conducted to assess the influence of the TyG index on MACE in the DM and no-DM groups and P for interaction was calculated.

The nonlinear association between TyG index as a continuous variable and MACE was evaluated using the adjusted RCS model. The variables in the model were consistent with the model 3. The number of knots was based on the lowest value of the Akaike information criterion and four knots were chosen for the analysis. Statistical analyses were performed using Stata version 15.0 software (4905 Lakeway Drive, College Station, Texas 77845 USA) and R software (R-project ^®^; R Foundation for Statistical Computing, Vienna, Austria, ver. 4.2.1). P < 0.05 was considered statistically significant.

## 3 Results

### 3.1 Subjects and baseline characteristics

2055 participants were included (**Figure 1**). **Table 1** showed the baseline characteristics among different TyG quartiles. Higher TyG levels were associated with younger age, higher heart rate, higher body mass index, and more comorbid conditions such as hypertension and diabetes. White blood cell, red blood cell, platelet, hemoglobin, triglyceride, ALT(Alanine Transaminase), albumin, creatinine, TC, LDL-C, uric acid, and HbA1c levels tended to rise as TyG quartiles rose, while HDL-C and BNP(B-natriuretic peptide) tended to decrease. In addition, patients in higher TyG quartiles received more beta-blockers, loop diuretics, metformin, alpha−glucosidase inhibitor, and insulin treatment.

**Table 1:** Characteristics of patients stratified by TyG quartiles.

Characteristics	Total (n=2055)	Quartiles of TyG				P Value
		Quartile 1 (n=514) TyG <8.54	Quartile 2 (n=514) 8.54≤TyG <8.93	Quartile 3 (n=514) 8.93≤TyG <9.41	Quartile 4 (n=513) TyG≥9.41	
**Age (years)**	60.3 ± 11.0	62.7 ± 10.6	60.2 ± 11.4	59.9 ± 10.7	58.3 ± 11.0	<0.001
**Sex, n (%)**						0.137
Male	1690 (82.2)	433 (84.2)	425 (82.7)	427 (83.1)	405 (79.0)	
Female	365 (17.8)	81 (15.8)	89 (17.3)	87 (16.9)	108 (21.0)	
**Vital signs**						
Systolic blood pressure(mmHg)	122.0 ± 18.2	122.3 ± 18.0	120.3 ± 17.8	123.5 ± 18.9	121.8 ± 17.9	0.047
Diastolic blood pressure(mmHg)	74.1 ± 13.1	74.0 ± 13.5	73.2 ± 13.0	75.0 ± 13.2	74.3 ± 12.7	0.180
Heart rate(beats/min)	73.8 ± 10.9	72.6 ± 10.1	73.4 ± 10.7	73.7 ± 10.7	75.5 ± 11.7	<0.001
Body mass index(kg/m^2^)	25.9 ± 3.2	25.1 ± 3.3	25.8 ± 3.1	26.4 ± 3.1	26.4 ± 3.2	<0.001
**NYHA class, n (%)**						0.405
I	225 (11.0)	48 (9.3)	65 (12.7)	54 (10.5)	58 (11.3)	
II	1073 (52.2)	268 (52.1)	263 (51.2)	268 (52.1)	274 (53.4)	
III	682 (33.2)	184 (35.8)	166 (32.3)	176 (34.2)	156 (30.4)	
IV	75(3.7)	14 (2.7)	20 (3.9)	16 (3.1)	25 (4.9)	
**Comorbidities, n (%)**						
Atrial fibrillation	86 (4.2)	30 (5.8)	18 (3.5)	18 (3.5)	20 (3.9)	0.187
Hypertension	1186 (57.7)	276 (53.7)	290 (56.4)	325 (63.2)	295 (57.5)	0.017
Diabetes	792 (38.5)	114 (22.2)	139 (27.0)	215 (41.8)	324 (63.2)	<0.001
Hypercholesterolemia	1498 (72.9)	373 (72.6)	368 (71.6)	381 (74.1)	376 (73.3)	0.825
Renal insufficiency	856 (41.7)	219 (42.6)	213 (41.4)	218 (42.4)	206 (40.2)	0.867
**History, n (%)**						
Prior stroke	179 (8.7)	55 (10.7)	39 (7.6)	43 (8.4)	42 (8.2)	0.303
Prior MI	505 (24.6)	130 (25.3)	121 (23.5)	128 (24.9)	126 (24.6)	0.925
Prior PCI	229 (11.1)	51 (9.9)	66 (12.8)	54 (10.5)	58 (11.3)	0.477
**Laboratory parameters**						
White blood cell (10^9^/L)	9.2 ± 5.1	8.7 ± 5.1	9.1 ± 5.0	8.9 ± 4.7	9.8 ± 5.4	0.002
Red blood cell (10^9^/L)	4.5 ± 0.6	4.4 ± 0.6	4.5 ± 0.5	4.6 ± 0.5	4.6 ± 0.6	<0.001
Platelet (10^9^/L)	235.8 ± 79.0	229.1 ± 81.4	237.9 ± 78.1	232.5 ± 74.6	243.6 ± 81.0	0.019
Hemoglobin (g/dL)	13.9 ± 1.8	13.6 ± 1.7	13.8 ± 1.7	14.0 ± 1.7	14.0 ± 1.9	<0.001
FBG (mg/dL)	131.0 ± 54.1	97.8 ± 18.7	110.8 ± 28.1	131.5 ± 41.3	183.9 ± 67.7	<0.001
Triglyceride (mg/dL)	150.7 ± 85.7	80.5 ± 20.7	119.2 ± 27.4	156.1 ± 42.2	247.0 ± 105.9	<0.001
ALT (U/L)	37.7 ± 37.8	30.5 ± 27.2	36.6 ± 35.2	40.5 ± 40.2	43.3 ± 45.3	<0.001
AST (U/L)	45.4 ± 54.9	41.1 ± 50.8	48.1 ± 59.0	43.7 ± 51.1	48.8 ± 58.2	0.078
Albumin (g/L)	41.6 ± 4.0	40.9 ± 3.9	41.8 ± 3.9	42.1 ± 3.8	41.8 ± 4.3	<0.001
Creatinine (mg/dL)	0.95 ± 0.33	0.90 ± 0.29	0.92 ± 0.30	0.98 ± 0.37	0.99 ± 0.36	<0.001
Blood nitrogen urea (mg/dL)	18.1 ± 7.8	17.7 ± 7.6	17.9 ± 8.0	18.2 ± 7.5	18.7 ± 8.1	0.216
eGFR (mL/min×1.73 m^2^)	87.6 ± 21.0	87.9 ± 19.9	87.8 ± 21.5	86.4 ± 22.3	88.4 ± 20.5	0.458
TC (mg/dL)	157.2 ± 40.9	143.9 ± 36.8	154.0 ± 37.5	160.2 ± 40.7	170.8 ± 43.5	<0.001
LDL-C (mg/dL)	94.1 ± 35.3	86.4 ± 33.1	93.9 ± 33.6	97.6 ± 35.5	98.7 ± 37.9	<0.001
HDL-C (mg/dL)	38.8 ± 9.5	42.0 ± 10.1	39.5 ± 9.4	37.9 ± 8.9	36.1 ± 8.3	<0.001
Sodium (mmol/L)	139.0 ± 3.0	139.1 ± 3.3	139.1 ± 3.0	139.1 ± 2.9	138.6 ± 3.0	0.014
Potassium (mmol/L)	4.2 ± 0.4	4.1 ± 0.4	4.2 ± 0.5	4.2 ± 0.4	4.2 ± 0.4	0.520
Uric acid (μmol/L)	368.8 ± 99.8	350.4 ± 94.5	369.3 ± 97.4	377.6 ± 103.1	377.9 ± 101.7	<0.001
HbA1c (%)	6.8 ± 1.4	6.1 ± 0.9	6.4 ± 1.1	6.8 ± 1.3	7.8 ± 1.6	<0.001
BNP (pg/ml)	407.2 ± 399.2	452.9 ± 438.3	396.2 ± 390.6	379.0 ± 357.2	401.0 ± 404.2	0.020
TyG	9.0 ± 0.7	8.2 ± 0.2	8.7 ± 0.1	9.2 ± 0.1	9.9 ± 0.4	<0.001
**Echocardiography**						
Left atrial diameter (millimeter)	39.3 ± 5.1	39.2 ± 5.5	39.5 ± 5.0	39.3 ± 4.6	39.1 ± 5.1	0.658
LVDs (millimeter)	41.0 ± 7.8	41.4 ± 7.9	40.9 ± 7.5	41.2 ± 7.9	41.0 ± 7.9	0.800
LVDd (millimeter)	54.8 ± 7.2	55.2 ± 7.3	54.8 ± 6.9	54.7 ± 7.3	54.6 ± 7.3	0.647
LVEF (%)	40.6 ± 6.2	40.5 ± 6.2	40.6 ± 5.9	41.0 ± 6.1	40.1 ± 6.7	0.187
**Medication use, n (%)**						
Aspirin	2047 (99.6)	514 (100)	512 (99.6)	511 (99.4)	510 (99.4)	0.389
Clopidogrel	1653 (80.4)	424 (82.5)	404 (78.6)	423 (82.3)	402 (78.4)	0.171
Ticagrelor	401 (19.5)	90 (17.5)	109 (21.2)	91 (17.7)	111 (21.6)	0.187
Statins	2042 (99.4)	511 (99.4)	509 (99.0)	513 (99.8)	509 (99.2)	0.438
Ezetimibe	499 (24.3)	122 (23.7)	123 (23.9)	128 (24.9)	126 (24.6)	0.970
Oral anticoagulants	90 (4.4)	23 (4.5)	18 (3.5)	21 (4.1)	28 (5.5)	0.478
Warfarin	40 (2.0)	9 (1.8)	7(1.4)	12 (2.3)	12 (2.3)	0.605
Factor Xa inhibitors	31 (1.5)	12 (2.3)	5 (1.0)	6 (1.2)	8 (1.6)	0.288
Factor IIa inhibitors	19(0.9)	2 (0.4)	6 (1.2)	3 (0.6)	8 (1.6)	0.183
CCB	261 (12.7)	63 (12.3)	71 (13.8)	67 (13.0)	60 (11.7)	0.757
Beta-blockers	1241 (60.4)	287 (55.8)	303 (59.0)	310 (60.3)	341 (66.5)	0.005
ACEI	176 (8.6)	37 (7.2)	52 (10.1)	45 (8.8)	42(8.2)	0.404
ARB	236 (11.5)	51 (9.9)	56 (10.9)	63 (12.3)	66 (12.9)	0.445
Diuretics	1369 (66.6)	329 (64.0)	336 (65.4)	342 (66.5)	362 (70.6)	0.137
Loop diuretics	1183 (57.6)	277 (53.9)	286 (55.6)	301 (58.6)	319 (62.2)	0.04
Thiazide diuretics	107 (5.2)	22 (4.3)	28 (5.5)	24 (4.7)	33 (6.4)	0.42
Spironolactone	957 (46.6)	239(46.5)	238 (46.3)	229 (44.6)	251 (48.9)	0.572
Tolvaptan	66 (3.2)	18 (3.5)	20 (3.9)	10 (2.0)	18 (3.5)	0.296
Sacubitril/valsartan	689 (33.5)	176 (34.2)	164 (31.9)	165 (32.1)	184 (35.9)	0.484
Metformin	191 (9.3)	20 (3.9)	23 (4.5)	61 (11.9)	87 (17.0)	<0.001
Alpha−glucosidase inhibitor	156 (7.6)	20 (3.9)	19 (3.7)	47 (9.1)	70 (13.7)	<0.001
Sulfonylurea	43 (2.1)	9 (1.8)	5 (1.0)	11 (2.1)	18 (3.5)	0.037
Insulin	475 (23.1)	89 (17.3)	105 (20.4)	113 (22.0)	168 (32.8)	<0.001
**Angiographic data, n (%)**						
LM disease	374 (18.2)	91 (17.7)	106 (20.6)	92 (17.9)	85 (16.6)	0.383
Three−vessel disease	1174 (57.1)	274 (53.3)	288 (56.0)	295 (57.4)	317 (61.8)	0.118
Chronic total occlusion	566 (27.5)	136 (26.5)	130 (25.3)	148 (28.8)	152 (29.6)	0.372
Diffuse lesion	388 (18.9)	110 (21.4)	93 (18.1)	86 (16.7)	99 (19.3)	0.267
In-stent restenosis	87 (4.2)	23 (4.5)	25 (4.9)	18 (3.5)	21 (4.1)	0.734
SYNTAX score	21.9 ± 7.8	21.8 ± 7.5	21.6 ± 7.7	21.9 ± 8.4	22.2 ± 7.7	0.677
**Procedural results**						
Target vessel territory, n (%)						
LM	340 (16.6)	79 (15.4)	96 (18.7)	87 (16.9)	78 (15.2)	0.403
LAD	1557 (75.8)	399 (77.6)	388 (75.5)	377 (73.4)	393 (76.6)	0.419
LCX	1317 (64.1)	314 (61.1)	326 (63.4)	321 (62.5)	356 (69.4)	0.030
RCA	1421 (69.2)	355 (69.1)	349 (67.9)	351(68.3)	366 (71.4)	0.633
Complete revascularization, n (%)	1250 (60.8)	318 (61.9)	309(60.1)	296 (57.6)	327 (63.7)	0.218
Number of stents	3.4 ± 1.5	3.2 ± 1.5	3.3 ± 1.5	3.4 ± 1.5	3.4 ± 1.5	0.160

Continuous variables were presented as mean ± SD. Categorical variables were presented as number (percentage). P values were calculated using analysis of variance, Chi-square test to compare differences in variables between different TyG quartiles. NYHA, New York Heart Association; MI, Myocardial Infarction; PCI, Percutaneous Coronary Intervention; FBG, fasting blood glucose; ALT, Alanine Transaminase; AST, Aspartate Transaminase; eGFR, estimated glomerular filtration rate; TC, total cholesterol; LDL-C, low-density lipoprotein cholesterol; HDL-C, high-density lipoprotein cholesterol; HbA1c, glycosylated hemoglobin A1c; BNP, B-natriuretic peptide; TyG, triglyceride-glucose index; LVDs, Left ventricular end systolic diameter; LVDd, Left ventricular end diastolic diameter; LVEF, left ventricular injection fraction; CCB, calcium channel blocker; ACEI, angiotensin-converting enzyme inhibitor; ARB, angiotensin receptor blocker; SYNTAX, synergy between PCI with taxus and cardiac surgery; LM, left main artery; LAD, left anterior descending artery; LCX, left circumflex artery; RCA, right coronary artery.

### 3.2 Associations between the TyG index and endpoints

The total incidence rate of MACE was 34.2%. As TyG quartiles increased, there was a significant increase in MACE (Quartile 4 *vs* Quartile 1: 48.3% *vs* 21.8%, P <0.001). The total rates of all-cause mortality, non-fatal MI, and any revascularization were 16.4%, 3.5%, and 14.4%, respectively. And the rates of all-cause mortality (P <0.001), non-fatal MI (P=0.044), and any revascularization (P <0.001) significantly increased as the TyG quartiles increased (**Table 2**).

**Table 2:** Outcomes of patients stratified by TyG quartiles.

Outcomes	Total (n=2055)	Quartiles of TyG				P Value
		Quartile 1 (n=514) TyG <8.54	Quartile 2 (n=514) 8.54≤TyG <8.93	Quartile 3 (n=514) 8.93≤TyG <9.41	Quartile 4 (n=513) TyG≥9.41	
MACE, n (%)	703 (34.2)	112 (21.8)	147 (28.6)	196 (38.1)	248 (48.3)	<0.001
All-cause mortality	337 (16.4)	52 (10.1)	63 (12.3)	92 (17.9)	130 (25.3)	<0.001
Non-fatal MI	71 (3.5)	11 (2.1)	13 (2.5)	25(4.9)	22 (4.3)	0.044
Any revascularization	295 (14.4)	49 (9.5)	71 (13.8)	79 (15.4)	96 (18.7)	<0.001

Categorical variables were presented as number (percentage). TyG, triglyceride-glucose index; MACE, Major Adverse Cardiovascular Events; MI, Myocardial Infarction.

**Figure 2:**
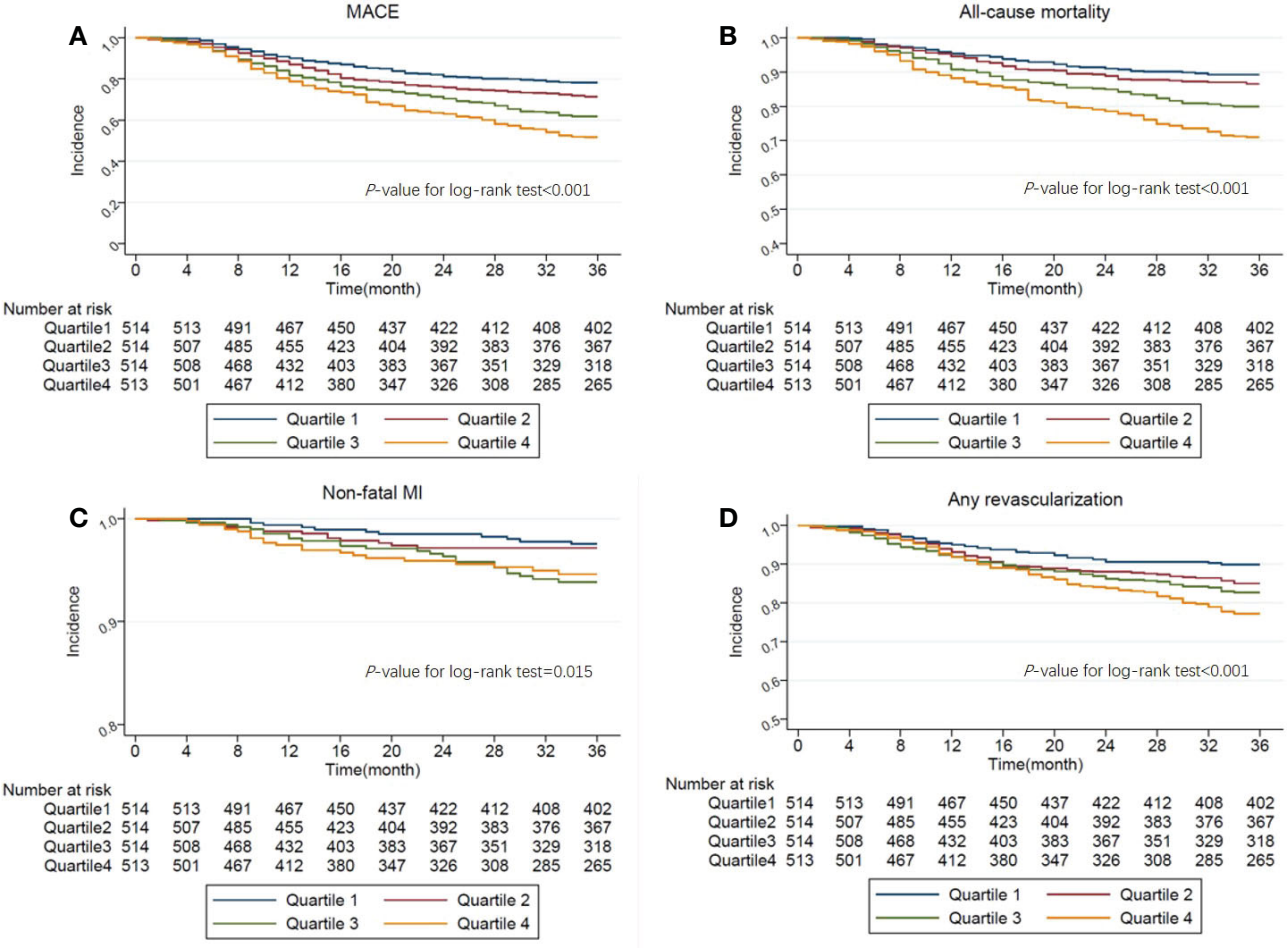
**(A)**. Kaplan-Meier curves showing the association between the TyG quartiles and MACE. **(B)**. Kaplan-Meier curves showing the association between the TyG quartiles and All-cause mortality. **(C)**. Kaplan-Meier curves showing the association between the TyG quartiles and Non-fatal MI. **(D)**. Kaplan-Meier curves showing the association between the TyG quartiles and Any revascularization.

The survival curves of MACE (Log-rank, P<0.001), all-cause mortality (Log-rank, P<0.001), non-fatal MI (Log-rank, P=0.0149), and any revascularization (Log-rank, P<0.001) stratified by the quartiles of TyG were shown in **Figure 2**, which demonstrated a significantly increased incidence of endpoints in patients as TyG quartiles increased.

The independent effect of TyG on MACE, all-cause mortality, and any revascularization was confirmed by Cox regression models. In the unadjusted model (model 1), a higher risk of MACE, all-cause mortality, non-fatal MI, and any revascularization was confirmed in higher quartiles of TyG. A higher TyG index was also proved to be correlated with the increased risk of MACE and other secondary outcomes when analyzed as a continuous variable in model 1. In model 2, age and sex were incorporated. The highest risk of MACE, all-cause mortality, non-fatal MI, and any revascularization was confirmed in the highest TyG quartile. When the TyG index was examined as a continuous variable in model 2, the results were consistent with the model 1. In model 3, more possible confounding variables were incorporated and the TyG index was still independently associated with the increased risk of MACE (Quartile 4 *vs* Quartile 1: HR, 95% CI: 1.92, 1.48-2.49; P<0.001, P for trend <0.001), all-cause mortality (HR, 95% CI: 2.18, 1.50-3.18; P<0.001, P for trend <0.001) and any revascularization (HR, 95% CI: 1.64, 1.09-2.46; P=0.017, P for trend =0.020), while there was no obvious correlation between TyG and non-fatal MI (HR, 95% CI: 1.94, 0.83-4.54; P=0.129, P for trend=0.047). When analyzed as a continuous variable in model 3, each unit higher TyG was still independently associated with the increased risk of MACE (HR, 95% CI: 1.41, 1.22-1.62; P<0.001), all-cause mortality (HR, 95% CI: 1.47, 1.20-1.80; P<0.001), non-fatal MI (HR, 95% CI: 1.62, 1.04-2.53; P=0.033) and any revascularization (HR, 95% CI: 1.30, 1.04-1.62; P=0.021). (**Table 3**).

**Table 3:** The association between TyG and outcomes.

	Model 1			Model 2			Model 3		
	HR (95% CIs)	P	P for trend	HR (95% CIs)	P	P for trend	HR (95% CIs)	P	P for trend
MACE			<0.001			<0.001			<0.001
Quartile 1: TyG <8.54	1.0 (Ref)			1.0 (Ref)			1.0 (Ref)		
Quartile 2: 8.54≤TyG <8.93	1.37 (1.07-1.76)	0.011		1.45 (1.13-1.85)	0.003		1.31 (1.02-1.68)	0.036	
Quartile 3: 8.93≤TyG <9.41	1.93 (1.53-2.44)	<0.001		2.06 (1.63-2.60)	<0.001		1.71 (1.34-2.18)	<0.001	
Quartile 4: TyG≥9.41	2.59 (2.07-3.24)	<0.001		2.88 (2.30-3.61)	<0.001		1.92 (1.48-2.49)	<0.001	
Continuous	1.82 (1.56-2.12)	<0.001		1.80 (1.62-2.00)	<0.001		1.41 (1.22-1.62)	<0.001	
All-cause mortality			<0.001			<0.001			<0.001
Quartile 1: TyG <8.54	1.0 (Ref)			1.0 (Ref)			1.0 (Ref)		
Quartile 2: 8.54≤TyG <8.93	1.27 (0.88-1.83)	0.203		1.33 (0.92-1.93)	0.124		1.24 (0.85-1.80)	0.270	
Quartile 3: 8.93≤TyG <9.41	1.95 (1.39-2.75)	<0.001		2.07 (1.47-2.92)	<0.001		1.78 (1.24-2.54)	0.002	
Quartile 4: TyG≥9.41	2.93 (2.12-4.04)	<0.001		3.23 (2.33-4.48)	<0.001		2.18 (1.50-3.18)	<0.001	
Continuous	3.22 (1.71-6.07)	<0.001		1.90 (1.63-2.22)	<0.001		1.47 (1.20-1.80)	<0.001	
Non-fatal MI			0.004			0.001			0.047
Quartile 1: TyG <8.54	1.0 (Ref)			1.0 (Ref)			1.0 (Ref)		
Quartile 2: 8.54≤TyG <8.93	1.24 (0.56-2.77)	0.601		1.35 (0.60-3.02)	0.465		1.21 (0.53-2.76)	0.643	
Quartile 3: 8.93≤TyG <9.41	2.52 (1.24-5.12)	0.011		2.79 (1.37-5.69)	0.005		2.46 (1.15-5.26)	0.020	
Quartile 4: TyG≥9.41	2.36 (1.14-4.87)	0.020		2.81 (1.35-5.85)	0.006		1. 94 (0.83-4.54)	0.129	
Continuous	1.74 (1.24-2.43)	0.001		1.89 (1.35-2.64)	<0.001		1.62 (1.04-2.53)	0.033	
Any revascularization			<0.001			<0.001			0.020
Quartile 1: TyG <8.54	1.0 (Ref)			1.0 (Ref)			1.0 (Ref)		
Quartile 2: 8.54≤TyG <8.93	1.52 (1.05-2.18)	0.025		1.59 (1.10-2.29)	0.013		1.39 (0.96- 2.02)	0.082	
Quartile 3: 8.93≤TyG <9.41	1.78 (1.25-2.54)	0.002		1.88 (1.32-2.69)	0.001		1.50 (1.03-2.19)	0.034	
Quartile 4: TyG≥9.41	2.29 (1.63-3.24)	<0.001		2.52 (1.77-3.57)	<0.001		1.64 (1.09-2.46)	0.017	
Continuous	1.60 (1.36-1.89)	<0.001		1.67 (1.41-1.97)	<0.001		1.30 (1.04-1.62)	0.021	

Models were derived from Cox proportional hazards regression analysis. Model 1: unadjusted. Model 2: adjusted for age, sex. Model 3: adjusted for age, sex, heart rate, body mass index, NYHA class, prior PCI, platelet, albumin, TC, LDL-C, HDL-C, potassium, uric acid, LVEF, ARB, thiazide diuretics, spironolactone, sacubitril/valsartan, diffuse lesion, SYNTAX score, LM disease, in-stent restenosis, target vessel (LM), complete revascularization. Abbreviation, NYHA, New York Heart Association; MI, Myocardial Infarction; PCI, Percutaneous Coronary Intervention; TC, total cholesterol; LDL-C, low-density lipoprotein cholesterol; HDL-C, high-density lipoprotein cholesterol; TyG, triglyceride-glucose index; LVEF, left ventricular injection fraction; ARB, angiotensin receptor blocker; SYNTAX, synergy between PCI with taxus and cardiac surgery; LM, left main artery; MACE, Major Adverse Cardiovascular Events; HR, hazards ratio; CI, confidence interval.

**Figure 3:**
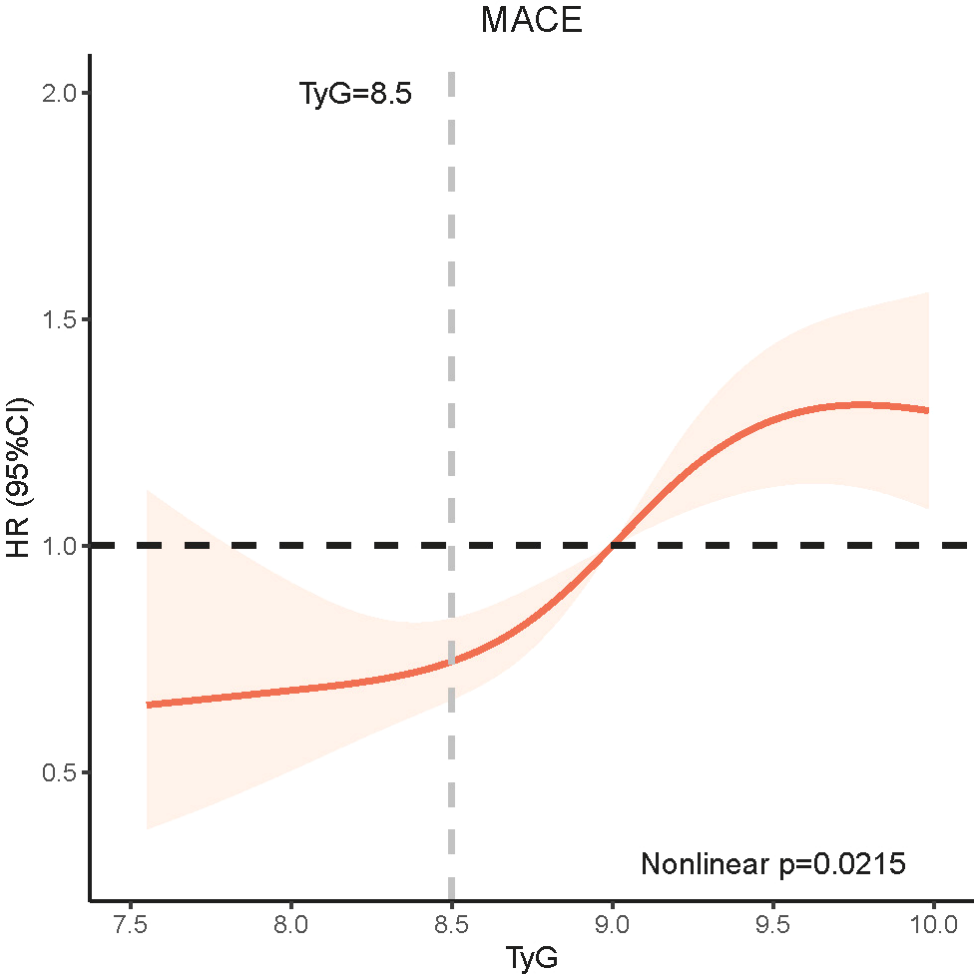
RCS model showing the association between the TyG quartiles and MACE.

RCS was conducted to fit model and visualize the relationship of MACE with TyG. RCS presented a nonlinear association between TyG index and MACE after adjusting for possible confounders (Nonlinear P=0.0215), though the risk increased as the TyG index increased in general (**Figure 3**).

### 3.3 Subgroup analysis

After adjusting for possible confounding variables, increased TyG quartiles were still correlated with the increased risk of MACE independently in both DM and no-DM groups. No obvious interaction was observed in the association of TyG and MACE between the DM and no-DM group (P for interaction=0.534) (**Table 4**).

**Table 4:** The association between TyG and MACE in patients with DM or no-DM.

DM	HR (95% CI)	P Value	No-DM	HR (95% CI)	P Value	P for interaction
Model 1			Model 1			0.951
Quartile 1: TyG<8.54	Reference		Quartile 1: TyG<8.54	Reference		
Quartile 2: 8.54≤TyG<8.93	1.22 (0.74, 2.02)	0.431	Quartile 2: 8.54≤TyG<8.93	1.43 (1.08, 1.89)	0.014	
Quartile 3: 8.93≤TyG<9.41	1.72 (1.10, 2.68)	0.016	Quartile 3: 8.93≤TyG<9.41	2.06 (1.56, 2.71)	<0.001	
Quartile 4: TyG≥9.41	2.48 (1.64, 3.75)	<0.001	Quartile 4: TyG≥9.41	2.62(1.95, 3.52)	<0.001	
Continuous	1.72 (1.46, 2.02)	<0.001	Continuous	1.80 (1.53, 2.12)	<0.001	
Model 2			Model 2			0.957
Quartile 1: TyG<8.54	Reference		Quartile 1: TyG<8.54	Reference		
Quartile 2: 8.54≤TyG<8.93	1.20 (0.73, 1.98)	0.475	Quartile 2: 8.54≤TyG<8.93	1.52 (1.15, 2.03)	0.004	
Quartile 3: 8.93≤TyG<9.41	1.73 (1.11, 2.71)	0.015	Quartile 3: 8.93≤TyG<9.41	2.22 (1.67, 2.93)	<0.001	
Quartile 4: TyG≥9.41	2.68 (1.77, 4.07)	<0.001	Quartile 4: TyG≥9.41	2.88 (2.13, 3.88)	<0.001	
Continuous	1.80 (1.54, 2.13)	<0.001	Continuous	1.87 (1.59, 2.20)	<0.001	
Model 3			Model 3			0.832
Quartile 1: TyG<8.54	Reference		Quartile 1: TyG<8.54	Reference		
Quartile 2: 8.54≤TyG<8.93	1.32 (0.79, 2.22)	0.290	Quartile 2: 8.54≤TyG<8.93	1.30 (0.97, 1.74)	0.084	
Quartile 3: 8.93≤TyG<9.41	1.75 (1.10, 2.79)	0.018	Quartile 3: 8.93≤TyG<9.41	1.70 (1.25, 2.30)	0.001	
Quartile 4: TyG≥9.41	2.25 (1.42, 3.59)	0.001	Quartile 4: TyG≥9.41	2.94 (1.20, 2.45)	0.003	
						
Continuous	1.61 (1.28, 2.01)	<0.001	Continuous	1.33 (1.08, 1.63)	0.007	

Subgroup analysis by diabetes mellitus (yes or no) of the association between the baseline triglyceride–glucose index and the MACE. Models were derived from Cox proportional hazards regression analysis. Model 1: unadjusted. Model 2: adjusted for age, sex. Model 3: adjusted for age, sex, heart rate, body mass index, NYHA class, prior PCI, platelet, albumin, TC, LDL-C, HDL-C, potassium, uric acid, LVEF, ARB, thiazide diuretics, spironolactone, sacubitril/valsartan, diffuse lesion, SYNTAX score, LM disease, in-stent restenosis, target vessel (LM), complete revascularization. NYHA, New York Heart Association; MI, Myocardial Infarction; PCI, Percutaneous Coronary Intervention; TC, total cholesterol; LDL-C, low-density lipoprotein cholesterol; HDL-C, high-density lipoprotein cholesterol; TyG, triglyceride-glucose index; LVEF, left ventricular injection fraction; ARB, angiotensin receptor blocker; SYNTAX, synergy between PCI with taxus and cardiac surgery; LM, left main artery; MACE, Major Adverse Cardiovascular Events; HR, hazards ratio; CI, confidence interval.

## 4 Discussion

We retrospectively evaluated the prognostic significance of IR assessed by the TyG index in ischemic HF participants undergoing PCI. Major findings were observed as follows: (1) Patients with higher TyG index had a higher incidence of MACE. (2) The independent correlation between the TyG index and the increased risk of MACE was confirmed. (3) The RCS analysis confirmed a nonlinear correlation between the TyG index and MACE. (4) According to subgroup analysis, no obvious interaction was observed in the association of the TyG index and MACE between the DM group and the no-DM group.

HF remains a major cause of mortality worldwide. Ischemia was thought to be the most important risk factor for HF, with approximately two-thirds of cases caused by CAD (coronary artery disease). Increasing frequency and duration of ischemic events lead to maladaptive remodeling of cardiomyocytes and expansion of the extracellular matrix, resulting in dilation of the cavity and systolic dysfunction (22). Moreover, the prevalence of HF has risen owing to the improved survival of patients after MI, which leads to further expansion of patients with ischemic HF (23).

IR has been proven to be tightly associated with the probability of CAD (24). Atherogenesis and plaque progression can be promoted by IR, the mechanisms of which likely involves more than just systemic factors, such as hyperlipidemia, high blood pressure, and a pro-inflammatory state. In addition, atherosclerosis may also be caused by a disruption in insulin signaling transduction among vascular intimal cells including endothelium, phagocytes, and SMC (25). Furthermore, a higher level of IR increases the risk of developing HF in individuals with or without DM (22, 26). The underlying reason may be that IR can result in the development of cardiac dysfunction through a series of molecular mechanisms, including dysregulated myocardial-endothelial interactions, mitochondrial dysfunction, oxidative stress, impaired calcium signaling, changes in substrate metabolism, and endoplasmic reticulum stress (27).

In previous studies, the TyG index has been identified as an effective marker of IR (28). In comparison to the hyperinsulinemic-euglycemic clamp technique, TyG is more convenient and accessible (13, 29, 30). It has recently been demonstrated that patients with a higher TyG index were more likely to suffer from diabetes and hypertension (31, 32). Moreover, a lot of clinical studies have shown that an increased TyG index was positively correlated with adverse prognosis in patients with ASCVD. An analysis of 1282 patients with stable CAD indicated that a higher TyG index was tightly associated with the increased risk of MACCE (major adverse cardiovascular and cerebral events) (16). Similarly, another retrospective cohort study involving 2531 patients with ACS (acute coronary syndrome) complicated by T2DM found that the TyG index was an independent predictor of MACE (14). As reported by Luo et al., the TyG index was correlated with MACCE positively in patients with STEMI following PCI (15). Furthermore, in patients with NSTE-ACS, the TyG index was also proved to be an independent predictor of a high SYNTAX score and MACE (17). The present study reached similar conclusions to those mentioned above, which may be related to the ability of the TyG index to cause coronary atherosclerosis as well as calcification (33, 34). Additionally, ACS patients undergoing PCI with an elevated TyG index have been shown to have a positive association with in-stent restenosis (35). These studies may explain why patients with high TyG are at high risk of revascularization.

The TyG index has also been found to be closely related to other indicators of CVD risk such as atrial stiffness and MINOCA (nonobstructive coronary arteries) (36–40). In a study by Huang et al., a higher TyG index was proved to be related to an increased risk of HF and impaired left ventricular structure and function (41). The study by Xu et al. also came to a similar conclusion that patients with the highest quartile of TyG index had a 24% higher risk of HF than those in the lowest quartile group of TyG index (42). In patients with established HF, a higher TyG index was found to be significantly correlated with a worse prognosis, according to Yang et al. They also demonstrated that among HF patients, TyG index could serve as a novel biomarker of myocardial fibrosis as well as a useful risk stratification metric in the management of HF (43). Among patients with chronic HF and diabetes, Guo et al. found a significant correlation between the TyG index and the prognosis. They revealed that cardiovascular mortality or rehospitalization due to HF was more likely to occur in higher TyG index groups (44).

As far as we know, this is the first study to investigate the prognostic value of the TyG index in ischemic HF patients undergoing PCI. In this study, TyG index was proved to be a reliable predictor of adverse prognosis in patients with ischemic HF who underwent PCI, which implied TyG index could be utilized in clinical practice as a predictor and had a positive effect on more comprehensive risk evaluation and stratification on the basis of traditional risk factors in this selected population.

Our findings of the subgroup analysis revealed that both diabetics and non-diabetics had a higher likelihood of MACE when the TyG index was higher, which was consistent with the previous study (41). This indicated that both diabetics and non-diabetics could benefit from the routine use of TyG index to assess insulin resistance.

We also found that when the TyG index exceeded 8.5, the slope of the RCS curve increased significantly, indicating the point TyG equal to 8.5 may be valuable. Therefore, the situation when the TyG index exceeds 8.5 in ischemic HF patients, should be given enough attention by clinicians. At the same time, we also noticed that at the end of the curve, the HR of MACE showed a downward trend, which may result from the bias caused by the insufficient amount of data here. Hence it is very necessary to increase the sample size of the high TyG population for further study.

Meanwhile, several important limitations of our study should be acknowledged. (1) As a single-center, retrospective, observational study, causality cannot be established in this study and the results may be weakened by this limitation. A multi-center study involving a larger population will be required in order to confirm the findings presented here. (2) The changes in the TyG index during the follow-up period were not assessed. (3) Statin therapy and diabetes medications were administered to a proportion of participants before or during admission, which may influence the TyG index. (4) All study participants are Chinese. Additional research is required to determine whether the results of the present study are applicable to other ethnic groups. (5) A comparison between the TyG index and hyperinsulinaemic-euglycaemic clamp test was not provided in the current study. (6) The data regarding the LVEF during the follow-up period were not assessed.

## 5 Conclusions

In patients with ischemic HF undergoing PCI, the TyG index, which can be easily measured and applied in clinical practice, contributed significantly to a higher risk of MACE. Prospective, randomized studies are required to determine whether interventions for IR could improve clinical prognosis.

## Data availability statement

The raw data supporting the conclusions of this article will be made available by the authors, without undue reservation.

## Ethics statement

Written or oral informed consent was obtained from each participant, and the study protocol was approved by the Clinical Research Ethics Committee of Beijing Anzhen Hospital, Capital Medical University.

## Author contributions

TS, XH contributed to this study equally. TS, XH and YZ contributed to study design, data collection, data analysis and manuscript writing. MM and BZ contributed to study design and intellectual direction. ZZ and ZC contributed to data collection and analysis. All authors contributed to the article and approved the submitted version.
