# Supplementary material for: Prognostic significance of the triglyceride-glucose index for patients with ischemic heart failure after percutaneous coronary intervention
Source: Front Endocrinol (Lausanne). 2023 Feb 6;14:1100399. doi: 10.3389/fendo.2023.1100399 (PMC9939475; doi:10.3389/fendo.2023.1100399)
Supplement: Supplementary file 1 [file Table_1.docx]

**Supplemental information (1)**

**1. Ischemic HF patients were diagnosed according to the following criteria:**

(1) HF diagnosis according to International Classification of Diseases (ICD) 10th revision I50.106 (left ventricular failure), I50.001 (congestive HF), I50.902 (Cardiac insufficiency), I50.919 (Diastolic heart failure HF), I50.905(Chronic HF) or I50.911 (HF, unspecified)

(2) concomitant multivessel disease (MVD) (coronary artery stenosis >50% in >_2 vessels or left main).

**2. The lesion characteristics of the coronary artery were defined as follows:**

(1) LM disease: an angiographically estimated stenosis >50% or a fractional flow reserve <0.80 in the left main coronary artery ostium, mid-shaft, or distal bifurcation.

(2) three‑vessel disease: more than two main coronary branches (vessel diameter ≥ 2 mm) with extent of stenosis ≥ 50%.

(3) chronic total occlusion lesion: lesion with complete obstruction [thrombolysis in myocardial infarction (TIMI) flow grade 0] lasting longer than 3 months, which was judged from the previous medical history or coronary angiogram results.

(4) diffuse lesion: a single stenotic lesion with a length of ≥ 20 mm.

(5) in-stent restenosis: stenosis of ≥ 50% occurring in the quantified by the synergy between PCI with taxus and cardiac surgery (SYNTAX) score.

**Supplemental information (2)**

Demographics included age, and sex. Vital signs included systolic blood pressure, diastolic blood pressure, heart rate, and body mass index. Comorbidities and medical history included atrial fibrillation, hypertension, diabetes mellitus, hypercholesterolemia, renal insufficiency, prior stroke, prior MI, and prior PCI. Laboratory parameters included white blood cell, red blood cell, platelet, hemoglobin, FBG (fasting blood glucose), triglyceride, ALT (Alanine Transaminase), AST (Aspartate Transaminase), albumin, creatinine, blood nitrogen urea, eGFR, TC (total cholesterol), LDL-C (low-density lipoprotein cholesterol), HDL-C (high-density lipoprotein cholesterol), sodium, potassium, uric acid, HbA1c (glycosylated hemoglobin A1c) and BNP (B-natriuretic peptide). Echocardiography data included left atrial diameter, LVDs (Left ventricular end systolic diameter), LVDd (Left ventricular end diastolic diameter) and LVEF (left ventricular injection fraction). Medication included aspirin, clopidogrel, ticagrelor, statins, ezetimibe, oral anticoagulants, warfarin, factor Xa inhibitors, factor IIa inhibitors, CCB (calcium channel blocker), beta-blockers, ACEI (angiotensin-converting enzyme inhibitor), ARB (angiotensin receptor blocker), diuretics, loop diuretics, thiazide diuretics, spironolactone, tolvaptan, sacubitril/valsartan, metformin, alpha‑glucosidase inhibitor, sulfonylurea, and insulin. Procedural results included target vessel territory (LM, LAD, LCX, RCA), complete revascularization, and number of stents.
